# The Role of Pathogen-Secreted Proteins in Fungal Vascular Wilt Diseases

**DOI:** 10.3390/ijms161023970

**Published:** 2015-10-09

**Authors:** Mara de Sain, Martijn Rep

**Affiliations:** Molecular Plant Pathology, Swammerdam Institute for Life Sciences, University of Amsterdam, Amsterdam 1098XH, The Netherlands; E-Mail: m.desain@uva.nl

**Keywords:** vascular wilt fungi, secreted proteins, effectors, pathogenicity, virulence, avirulence

## Abstract

A limited number of fungi can cause wilting disease in plants through colonization of the vascular system, the most well-known being *Verticillium dahliae* and *Fusarium oxysporum*. Like all pathogenic microorganisms, vascular wilt fungi secrete proteins during host colonization. Whole-genome sequencing and proteomics screens have identified many of these proteins, including small, usually cysteine-rich proteins, necrosis-inducing proteins and enzymes. Gene deletion experiments have provided evidence that some of these proteins are required for pathogenicity, while the role of other secreted proteins remains enigmatic. On the other hand, the plant immune system can recognize some secreted proteins or their actions, resulting in disease resistance. We give an overview of proteins currently known to be secreted by vascular wilt fungi and discuss their role in pathogenicity and plant immunity.

## 1. Vascular Wilt Fungi

Only a few fungal species are able to colonize the plant vascular system and cause wilt disease. These include *Fusarium oxysporum* (*Fo*) and several species belonging to the genera *Verticillium*, *Ceratocystis* and *Ophiostoma* [1]. The symptoms associated with vascular wilt disease depend on the fungal species and the host plant, but generally include discoloration of the vessels, wilting, defoliation, stunting and plant death [1].

*Fo* is a soil-borne rhizosphere colonizer and, in specific strain-plant combinations, a xylem-colonizing fungus, causing vascular wilt disease. *Fo* produces several types of asexual spores, including chlamydospores, which can survive in the soil for many years [1]. Once the presence of plant roots is detected, germination into infection hyphae is initiated [2]. These hyphae attach to and colonize the root surface. They usually penetrate the roots through natural openings and do not require specialized infection structures like appressoria [2], although hyphal swelling has been observed at penetration points [2,3,4]. The fungus then grows in the root cortex, until it enters and colonizes the xylem vessels [2]. *Fo* is able to infect over a hundred plant species, ranging from vegetables to flowers to field and plantation crops [3]. However, single strains usually infect only one or a few plant species. Based on host-specificity, pathogenic strains of *Fo* are grouped into *formae speciales* (f. sp.). Genome sequencing of tomato-infecting *Fo* f. sp. *lycopersici* (*Fol*) strain 4287 identified the presence of lineage-specific (LS) regions, absent in *Fusarium graminearum* and *Fusarium verticilloides* [5]. These LS regions show different characteristics compared to the core genome, most prominently a very high density of transposable elements. Compared to the core genome the LS regions are much more divergent between different *formae specialis*, suggesting a role in host adaptation [5]. Furthermore, the transfer of LS chromosome 14 from *Fol*4287 to a non-pathogenic *Fo* strain turned the strain into a tomato pathogen, showing the importance of LS regions for pathogenicity [5]. Interestingly, genes for secreted proteins, especially small, in xylem secreted proteins, are enriched in LS regions [5,6].

Verticillium wilt occurs on many dicotyledonous plants. The primary causal agent is *Verticillium dahliae* (*Vd*), which has a very wide host range of over 200 plant species and is mainly found in temperate and subtropical regions [1,7]. Another causal agent of Verticillium wilt, *Verticillium albo-atrum* (*Vaa*), has a much narrower host range and thrives at temperatures around 21 °C [1]. Both *Vd* and *Vaa* are soil-borne, vascular fungi and their life cycle is in many ways similar to that of *Fo*. Germination of their fungal resting structures (sclerotia) in the soil can be induced by plant root exudate [1]. Hyphae can then grow a limited distance to reach the roots of a potential host plant and start penetration. Roots are usually penetrated at easily accessible sites, like root tips or at points of lateral root formation [1]. After crossing the endodermis the fungus enters the vascular tissue, usually through the pits. This entire process, from germination to entering the xylem vessels, takes around three days. After hyphae invade the xylem vessels, conidia are formed. These contribute to a faster spread of the pathogen, as they are carried in the xylem fluid. If conidia are trapped at pit cavities or at the end of a vessel, they can germinate into hyphae and penetrate a neighboring vessel, where they can sporulate again [1,7]. Conidiation may play an important role in virulence, as more heavily conidiating strains are more aggressive [7]. At the final stage of infection the fungus is no longer limited to the plant vascular system and starts to generate resting structures. *Vd* starts to produce microsclerotia that can survive for over a decade in the soil [1,7], similar to *Fo* chlamydiospores. The resting mycelium of *Vaa* has a shorter lifetime. However, *Vaa* can also produce air-borne conidia, as an alternative infection strategy [1,7]. Both *Vd* and *Vaa* genomes were sequenced. Compared to those of other fungi, *Vd* and *Vaa* genomes contain more genes for cell-wall degrading enzymes (CWDEs) [8]. Interestingly, the pectate lyase 11 family has only been identified in vascular wilt fungi [8], suggesting this family may be required for growth in the xylem. Overall, the *Vd* and *Vaa* genomes that were sequenced are very similar. However, the *Vd* genome is larger and contains four LS regions absent in *Vaa* [8]. These regions are repeat-rich, have a high density of transposable elements, vary substantially between *Vd* strains and are suspected to play an important role in pathogen adaptation and virulence [8,9]. However, unlike the *Fol* genome, secreted proteins are not enriched in these regions [9].

Three *Ophiostoma* species can cause wilting disease on elm trees, known as Dutch elm disease. The first is *Ophiostoma ulmi* (*Ou*), which caused an outbreak of Dutch elm disease in Western Europe in the early 1900s and later spread to North America [1]. The second is *Ophiostoma novo-ulmi* (*Onu*), which caused a second pandemic and is more virulent than *Ou* [10]. The third species was found in the western Himalayas and named *Ophiostoma himal-ulmi* (*Ohu*) [11]. While no disease symptoms were observed on the Himalayan elm trees from which the species was isolated, infection assays have shown that it can cause vascular wilt symptoms on susceptible elm trees, to a similar extent as *Onu* [11]. While *Fo*, *Vd* and *Vaa* are soil-born, the *Ophiostoma* species that cause wilting disease on elm trees are mainly transmitted by bark beetles (*Scolytus* and *Hylurgopinus rufipes*) [1]. Therefore, disease is dependent not only on the interaction between fungus and plant, but also on the interaction between plant and beetle and between fungus and beetle. Bark beetles carrying fungal spores on their exoskeleton spread the disease when feeding on elm trees [1]. Pre-existing (feeding) wounds give the fungus direct access to the vascular tissue. Like *Verticillium*, fungal spores allow *Ophiostoma* to quickly spread through individual xylem vessels, while hyphae are able to penetrate neighboring vessels through pit membranes [1]. The fungus colonizes breeding and oviposition tunnels made by female beetles and produces sticky conidiophores, which can attach to the exoskeleton of young bark beetles that fly out to feed, starting a new infection cycle. The genomes of *Ou* and *Onu* have been sequenced and annotated [12,13,14]. Their size, 31.5 and 31.8 Mb, respectively, is similar to that of *Vd* and *Vaa*. The *Onu* genome counts 621 proteins with a predicted signal peptide, which is a relatively small number for fungi [14].

Most *Ceratocystis* species do not cause wilting disease. An exception is *Ceratocystis fagacearum* (*Cfag*), first identified as the causal agent of oak wilt in Wisconsin, USA, in 1942 [15]. Currently, the disease is found in Texas and many eastern and mid-western states in the USA [16]. There are large differences in susceptibility between different oak species. In general, white oaks are tolerant, while red oaks are highly susceptible and die within a year after infection [1,17]. The pathogen can spread in several ways [1,17]. Short distance transmission can be accomplished by natural root grafts between diseased and healthy trees, which makes stem density an important factor in disease incidence. Long distance transmission, also known as overland spread, is dependent on insect vectors. Under the right conditions *Cfag* can produce sporulation mats that emit “fruity” odors that, among others, attract sap-feeding nitidulid beetles [18]. Transmission can take place when nitidulid beetles carrying spores from these mats move on to feed on fresh wounds of uninfected oak trees [1,17]. *Cfag* enters oak trees through these fresh wounds and initially grows in the xylem vessels of the outer sapwood [19]. Later in the infection cycle, hyphae are formed that penetrate the parenchyma cells and grow inter- and intracellularly. During the final disease stage, when the tree is dying, sporulation mats are sometimes formed on red oak trees, forming a new primary infection source [17]. Besides *Cfag*, there are some other *Ceratocystis* species that cause wilt disease, for example, on mango, eucalyptus and cacao [20,21,22].

Pathogens, including vascular wilt fungi, secrete proteins during colonization to establish a successful pathogen-host interaction. In this review, we will give an overview of proteins secreted by vascular wilt fungi for which a role in virulence has been described (Table 1). These include small, usually cysteine-rich proteins, necrosis-inducing proteins, enzymes that target plant physical or chemical barriers and induced defense responses and saponins. Furthermore, we will discuss the recognition of some of these secreted proteins by the plant immune system.

**Table 1:** Pathogen-secreted proteins of vascular wilt fungi.

Protein Name	Pathogen	Virulence Phenotype Deletion Mutant	Avr ^1^	Comments	Reference
**Small, Secreted Proteins**					
Six1	*Fol*	reduced virulence on tomato	yes (*I-3*)		[23]
Six2	*Fol*				[23]
Six3	*Fol*	reduced virulence on tomato	yes (*I-2*)	Interacts with Six5	[23]
Six4	*Fol*	no suppression of *I-2*/*I-3*-mediated resistance on tomato	yes (*I*, *I-1*)		[23]
Six4	*Fo*5176	reduced virulence on *At*			[24]
Six4	*Foc*	reduced virulence on cabbage			[25]
Six5	*Fol*	reduced virulence on tomato		Interacts with Six3 Required for *I-2*-mediated immunity	[6]
Six6	*Fol*	reduced virulence on tomato			[6]
Six7	*Fol*				[6]
Six8	*Fol*			Multi-copy gene in Fol	[6]
Six8b	*Fol*			Multi-copy gene in Fol	[6]
Six9-14	*Fol*				[6]
VDAG_05180	*Vd Vd*Ls17	reduced virulence on tomato		Two LysM domains	[9]
Ave1	*Vd* JR2	reduced virulence on tomato	yes (*Ve1*)	Homology to PNPs	[26]
Ave1	*Fol*		yes (*Ve1*)	Not found in xylem during infection	[26]
XLOC_009059	*Vd* JR2	reduced virulence on tomato			[9]
XLOC_008951	*Vd* JR2	reduced virulence on tomato			[9]
**NEP (-like) Proteins**					
NEP1	*Foe*	no virulence effect on coca		Ethylene and necrosis-inducing factor on several dicotylous plants	[27,28]
NEP	*Vd*-8			Wilt-inducing factor on cotton	[29]
NLP1	*Vd* V592	no virulence effect on cotton		Wilt- and necrosis-inducing factor on cotton	[30]
NLP2	*Vd* V592	no virulence effect on cotton		Wilt- and necrosis-inducing factor on cotton	[30]
NLP3-9	*Vd* V592				[30]
NLP1	*Vd* JR2	reduced virulence on tomato, *At* and *Nb*		Necrosis-inducing factor on *Nb*; Mutant has reduced conidiophore formation and extensive formation of aerial hyphae	[31]
NLP2	*Vd* JR2	reduced virulence on tomato and *At*		Necrosis-inducing factor on *Nb*	[31]
NLP3-9	*Vd* JR2				[31]
**Secreted Enzymes**					
PG1	*Fol*	*Δpg1Δpgx6* double mutant reduced virulence on tomato			[32]
PGX6	*Fol*	*Δpg1Δpgx6* double mutant reduced virulence on tomato			[32]
TOM1	*Fol*	reduced virulence on tomato		Tomatinase activity ~25% reduced in deletion mutant	[33]
Mep1	*Fol*	*Δfomep1Δfosep1* double mutant reduced virulence on tomato			[34]
Sep1	*Fol*	*Δfomep1Δfosep1* double mutant reduced virulence on tomato			[34]
Isc1	*Vd* V991	reduced virulence on cotton			[35]
**Hydrophobins**					
Cerato-ulmin	*Onu*	no virulence effect on elm			[36]
VDH1	*Vd* Dvd-T5	no virulence effect on tomato		Reduced microsclerotia production in deletion mutant	[37]

Six = secreted in xylem; NEP = necrosis- and ethylene-inducing protein; NLP = NEP-like protein; PG = endopolygalacturonase; PGX = exopolygalacturonase; TOM = tomatinase; Mep = metalloprotease; Sep = serine protease; Isc = isochorismatase; *Fol* = *Fusarium oxysporum* f. sp. *lycopersici*; *Fo* = *Fusarium oxysporum*; *Foc* = *Fusarium oxysporum* f. sp. *conglutinans*; *Vd* = *Verticillium dahliae*; *Foe* = *Fusarium oxysporum* f. sp. *erythroxyli*; *Onu* = *Ophiostoma novo-ulmi*; *At* = *Arabidopsis thaliana*; *Nb* = *Nicotiana benthamiana*; Avr = avirulence; I = immunity; LysM = lysin motif; PNP = plant natriuretic peptide; ^1^ Corresponding R gene between brackets.

## 2. Small, Cysteine-Rich Proteins Secreted by *Fusarium oxysporum*

In total, the annotated genome of *Fol* strain 4287 encodes 126 small (less than 200 amino acids), cysteine-rich (minimum of four cysteines), potentially secreted proteins [5]. Research has mainly focused on a subset of these proteins that were identified in the xylem sap of infected tomato plants, named Secreted in xylem (Six) 1–14 [6,23]. All *SIX* genes are located in LS regions, most on chromosome 14 of strain 4287 [5,6]. Remarkably, they all have Miniature Inverted-repeat Transposable Elements (MITEs), which are non-autonomous transposable elements, in their upstream region [6]. While no *SIX* homologs have been identified in the *Vd* or *Vaa* genome [8], homologs are present in other *formae speciales* of *Fo* [24,38,39,40,41]. The presence and absence of individual *SIX* genes and sequence variation within *SIX* genes can be used to discriminate between different *formae specialis*, races and isolates [39,40,41].

The first small, cysteine-rich protein identified in xylem sap of *Fol*-infected tomato plants was named Six1 [42,43]. The protein is also known as Avirulence (Avr) 3, because it is recognized by the tomato Resistance (R) protein Immunity I-3 [44]. Expression of *SIX1* is strongly induced in the presence of living plant cells and requires the transcription factor Six gene expression 1 [45,46]. The full-length gene encodes a 32 kDa protein that contains eight cysteine-residues, a signal peptide and a prodomain [23,44]. The protein is required for full virulence, as tomato plants infected with a *SIX1* deletion strain show reduced disease symptoms [43]. How Six1 enhances virulence is currently unknown. Interaction screens have revealed that Six1 can interact with small heat-shock proteins [47]. However, it seems unlikely that Six1 has an intracellular effector target, as it is recognized outside the plant cell by the receptor-like kinase (RLK) resistance protein I-3 [48].

The *Fol SIX3* gene encodes an 18 kDa protein with a signal peptide and only two cysteine-residues [23]. The protein, Six3, is also referred to as Avr2, because it is recognized inside the plant cell by the intracellular tomato R protein I-2 [49]. Like Six1, Six3 is also required for full virulence on tomato plants [49]. While *SIX1* is already expressed during the early stages of root colonization, *SIX3* is mainly expressed during hyphal growth in the xylem vessels [45,50]. *Fol SIX3* shares its upstream sequence with *SIX5*, which encodes a 12 kDa mature protein that contains six cysteines [6,23]. Interestingly, Six3 is capable of forming homodimers with itself and heterodimers with Six5 [50,51]. Bimolecular fluorescence complementation assays have shown that Six3 homodimers localize to the nucleus and the cytoplasm, while Six3–Six5 heterodimers are present in the nucleus, the cytoplasm and in spots at the cell periphery [51]. It will be interesting to identify the nature of these spots, as this could give insight into the function of Six3 and Six5.

*SIX6* is present in *Fo* species infecting tomato, melon, watermelon, passion fruit, cucumber and cotton [39,41,52]. Homologs have also been found in two *Colletotrichum* species [52]. Recent RNA-sequencing analysis has shown that an intron in *Fol SIX6* was missed during earlier annotation and that the newly annotated gene encodes a 23 kDa mature protein containing eight cysteine residues [53]. Its gene product is required for full virulence, as tomato plants inoculated with *SIX6* deletion mutants have a higher plant weight, compared to wild-type inoculated plants [52]. Transient expression of *SIX6* without its signal peptide can suppress cell-death and ion leakage induced by the Avr2-I-2 pair in *N. benthamiana* (*Nicotiana benthamiana*) leaf cells [52]. This suggests that Six6 might be involved in suppression of defense responses, although in disease assays I-2-mediated resistance is unaffected by the presence of *SIX6* in the fungus [52].

*SIX8* is present in several *formae speciales*, including *Fo* f. sp. *cubense* (*Foc*), the causal agent of panama disease on banana plants, and *Fol* [6,40]. While most *SIX* genes in *Fol* are single-copy, *SIX8* is a multi-copy gene [6]. In the *Fol*4287 genome nine identical copies have been identified in LS and telomeric regions, and among *Fol* strains the copy number varies between three and 13 [54]. Furthermore, four copies of a homologous gene were identified in the *Fol*4287 genome and named *SIX8b* [6]. However, Six8b has never been identified in xylem sap of infected tomato plants, suggesting that the gene is not expressed during infection [6]. *SIX8* deletion strains have not been made, due to its multi-copy nature, and therefore it is unknown whether Six8 contributes to virulence.

Unlike most other Six proteins, *Fol* Six4 is not required for full virulence on susceptible tomato plants [55]. Instead, *SIX4* deletion and complementation experiments have shown that this effector can suppress both I-2- and I-3-mediated resistance, but not I-7-mediated resistance [55,56]. This is interesting, because I-2 and I-3 belong to two different R protein classes: I-2 is an intracellular R protein encoding a Coiled-Coil (CC)-Nucleotide-Binding (NB)-Leucine-Rich Repeat (LRR) protein [57], while I-3 is an S-RLK (SRLK) located on the plasma membrane [48]. I-7 belongs to yet another class and is a LRR-Receptor-Like Protein (RLP) [56]. This suggests that Six4 manipulates a process in tomato plants that is required for CC-NB-LRR and SRLK types of resistance proteins, but not for LRR-RLP-mediated resistance. As I-7-mediated resistance is dependent on the downstream signaling component Enhanced Disease Susceptibility 1 (EDS1) and CC-NB-LRR-mediated resistance is independent of EDS1, it has been suggested that Six4 can only suppress EDS1-independent resistance responses [56]. A complicating factor is the strain-specificity of the suppression effect; there are strains that contain *SIX4* but are unable to suppress I-2- and I-3-mediated resistance [58,59]. It was shown that the inability of Six4 to suppress resistance in these strains is not due to sequence differences in the gene, nor to changes in local genetic context, nor to alterations in *SIX4* expression, suggesting that another (unknown) fungal factor is involved [59]. Interestingly, Six4 is required for full virulence of an Arabidopsis-infecting *Fo* strain: infection assays with a *SIX4* deletion strain showed reduced disease symptoms and reduced fungal biomass compared to the wild-type strain *Fo*5176 [24]. Likewise, *SIX4* deletion in *Fo* f. sp. *conglutinans* resulted in reduced disease symptoms on both susceptible and resistant cabbage plants, compared to wild-type and *SIX4*-complemented strains [25]. Pull-down experiments with *Fol* Six4 as bait followed by mass spectrometry suggests that Six4 can interact with glutamate decarboxylase (our unpublished results). This enzyme is involved in the conversion of glutamate to gamma-aminobutyric acid (GABA). Interestingly, there are indications that GABA plays a role in the promotion of cell death [60,61,62]. Possibly, Six4 interferes with this process.

In summary, it has been demonstrated that several *Fo* Six proteins contribute to virulence and are therefore genuine effectors. How they do so is as yet unknown and the hope is that identification of plant proteins interacting with effectors, and plant processes perturbed by them, will provide clues to their function.

## 3. Small Proteins Secreted by *Verticillium* during Host Colonization

Candidate effectors are often identified by genome searches for small, cysteine-rich secreted proteins. The *Vd* and *Vaa* annotated genomes both count ~120 genes encoding hypothetical proteins that contain less than 400 amino acids and at least four cysteine-residues [8]. None of these are homologous to the *Fo* Six proteins. Analysis of the *Vd* and *Vaa* genomes did identify proteins that show homology to *Cladosporium fulvum* lysin motif (LysM) effectors [8,63]. The core *Vd* genome counts four putative LysM effector genes [64]. These core LysM effector genes do not seem to contribute to pathogenicity, as they are not expressed during infection on *N. benthamiana* or tomato plants and single gene deletions do not show altered virulence on tomato [9,64]. However, LysM effectors can act as virulence factors in *Vd*, as a strain-specific LysM effector gene (*VDAG_05180*), located in an LS region, is required for full disease development and host colonization [9]. LysM effectors have been implicated in suppressing chitin-triggered immune responses, either by protecting fungal hyphae against degradation by host chitinases or by sequestering cell wall-derived chitin fragments to prevent host detection [65,66,67,68]. VDAG_05180 may have a similar role, as the *in planta* produced protein can bind chitin and is able to suppress a chitin-induced pH shift in a tomato cell culture that is indicative of chitin-triggered immune responses [64]. LysM effector genes are also present in the *Fol*4287 genome, but have not been functionally characterized.

*Vd* strains that cause Verticillium wilt on tomato plants are divided into two races. Race 1 strains are recognized by the resistance gene *Ve1*, while race 2 strains are not [69,70]. Comparative genomics between *Vd* strains belonging to both races combined with RNA-sequencing identified *Avirulence on Ve1* (*Ave1*) in a 50-kb race 1-specific region [26]. *Vd Ave1* is induced during infection and encodes a secreted protein that is recognized by Ve1 [26]. Ave1 shows homology to plant natriuretic peptides (PNPs), suggesting the gene was acquired through horizontal transfer from plants [26]. While *Vd* Ave1 is an avirulence protein on tomato plants containing *Ve1*, infection assays have shown it is required for full virulence on susceptible tomato plants [26]. While there is no functional data on how Ave1 enhances virulence, it has been suggested that the protein affects water and ion homeostasis based on its homology to PNPs [26].

*Ave1* and the LysM effector *VDAG_05180* are both located in *Vd* LS regions [9]. Hence, it was hypothesized that other genes located in these regions also contribute to virulence. Two genes encoding secreted proteins, *XLOC_009059* and *XLOC_008951*, located in LS regions of *Vd* strain JR2 were chosen for gene deletion, because they are highly up-regulated during infection [9]. Pathogenicity assays on tomato plants indicate that these genes are indeed required for full virulence, as plants infected with deletion strains show an increase in canopy area and a reduction in fungal biomass compared to plants infected with wild-type JR2 [9]. The next step will be to find out how these proteins contribute to virulence at the molecular level.

## 4. Nep1 (-Like) Proteins

Two decades ago, a 24-kDa Necrosis and ethylene inducing peptide (Nep1) was isolated from *Fo* f. sp. *erythroxyli* (*Foe*) culture filtrate [27]. The protein causes cell death in dicots, but not in monocots [27,71,72,73]. *Fo* Nep1 is a member of a large family of proteins secreted by microbes, including plant pathogenic bacteria, oomycetes and fungi [74,75,76]. Proteins belonging to this family are collectively named Nep1-like proteins (NLPs), after the founding member. The family is characterized by a Necrosis-inducing *Phytophthora* Protein (NPP) domain, which contains a highly conserved heptapeptide motif: GHRHDWE [77]. Initially, the family was divided into two groups based on the number of cysteine-residues in the NPP domain [75]. Recently, a third, more divergent group was identified [76]. There is also functional diversification in this superfamily of proteins, as some members are cytotoxic, whereas others are not [30,78,79,80,81]. This cytotoxicity is only partially understood and could be due either to plant membrane disruption, induction of plant innate immune responses or a combination of both processes [82,83,84,85].

In the *Fol*4287 genome seven *NLP* family members were identified, three of which are located in LS regions [5]. None of the *Fo* NLPs have been functionally characterized, except for the cytotoxic *Foe* Nep1. Electron microscopy showed that spray application of the protein caused thinning of the cuticle and breakdown of chloroplasts in several plant species [73]. However, neither deletion nor overexpression of *NEP1* affected *Foe* pathogenicity on coca, suggesting the protein does not have a virulence function on coca plants [28].

In an experiment designed to identify potential elicitor proteins, the first *Vd* NLP, named *Vd* Nep, was identified by sequencing expressed sequence tags (ESTs) from the cotton-pathogenic Vd-8 strain [29]. The purified protein is cytotoxic; it induces necrosis in *Nicotiana benthamiana* and *Arabidopsis thaliana*. Infiltration of purified *Vd* Nep into *Arabidopsis* leaves induced defense responses, as expression of marker genes for ethylene biosynthesis, salicylic acid and jasmonic acid signaling was increased [29]. In cotton suspension cells, the purified protein was able to activate the formation of sesquiterpene aldehydes and programmed cell death [29]. Since then, it was shown that most *Vd* strains contain eight or nine NLP members, named *Vd* NLP1-9 [8,30,31]. Using this nomenclature, *Vd* NLP1 is the homolog of the initially identified *Vd* Nep [30]. The cytotoxicity of *Vd* NLP1-9 from the cotton-infecting strain V592 and from the tomato-infecting strain JR2 has been investigated. In both cases, only NLP1 and NLP2 induce cell death upon infiltration in *N. benthamiana* leaves [30,31]. Furthermore, both *Vd* NLP1 and *Vd* NLP2 from cotton-infecting isolates are able to produce wilt symptoms in cotton hypocotyls [29,30], suggesting they might be involved in symptom development. However, both single (*nlp1*, *nlp2*) and double (*nlp1*/*nlp2*) gene deletions in *Vd* strain V592 did not reduce symptom development on cotton plants compared to wild-type [30]. Targeted deletion of *NLP1* in strain JR2, on the other hand, negatively affected virulence on tomato, *Arabidopsis* and *Nicotiana benthamiana* plants [31]. Surprisingly, *NLP1* deletion strains showed a vegetative growth phenotype; they produced more aerial hyphae and less conidiophores, which could be reversed by re-introducing the wild-type gene [31]. Deletion of *NLP2* did not alter vegetative growth of strain JR2, but did reduce virulence on tomato and *Arabidopsis* [31]. Virulence on *Nicotiana benthamiana* plants was not altered, most likely because *NLP2* is not expressed during infection of this host plant [31].

Compared to other fungi, which usually contain only two or three NLPs, the NLP family is expanded in the wilt pathogens *Fo*, *Vd* and *Vaa*, [5,8]. It has been suggested that this expansion contributes to the broad host range of these fungi and/or to the development of their typical wilt symptoms. While a role in virulence has been shown for some of the cytotoxic NLPs in *Vd*, the non-cytotoxic NLPs from wilt pathogens have not yet been tested for their contribution to pathogenicity. Future research should aim to elucidate the exact function of single NLPs in this diverse protein family and show whether they play a specific role in wilt disease.

## 5. Enzymes Secreted by Wilt Fungi

Plant pathogens, including wilt fungi, secrete many enzymes that may contribute to virulence. These include enzymes that target plant physical barriers, chemical barriers and induced defense responses. One of these physical barriers is the plant cell wall, which can be broken down by cell-wall degrading enzymes (CWDEs). Comparative analysis of fungal genomes has shown that the highest numbers of carbohydrate-active enzymes are generally found in plant-pathogenic fungi [86]. Furthermore, targeted deletion of genes involved in the induction of CWDEs in *Fo* and *Vd* resulted in reduced fungal colonization [87,88], suggesting CWDEs are important for pathogenicity. Although we will not discuss CWDEs of vascular wilt fungi in depth, we will give an example that shows that at least some CWDEs are virulence factors.

The *Fol*4287 genome encodes four endopolygalacturonases (PGs) and four exopolygalacturonases (PGXs), which are all pectin-degrading enzymes [32]. Assays with mutant strains showed that *PG1* and *PGX6* contribute most to secreted PG activity [32]. Hence, these mutant strains were used for infection assays. While infection with single gene deletion strains only marginally delayed plant death, infection with a double *PG1*/*PG6* deletion strain resulted in clearly reduced plant mortality [32]. Apparently, PG1 and PG6 each have an activity (presumably pectin degradation) that is required for full *Fol* virulence.

Plants also contain chemical barriers for protection against microbes, for example saponins. Saponins are plant glycosides with soap-like properties. The major saponin in tomato is alpha-tomatine, which shows anti-fungal activity [89,90]. This activity has been ascribed to its ability to bind sterols in fungal membranes, creating a (transient) loss of membrane integrity followed by cellular leakage [89,91,92]. More recently, however, it was shown that alpha-tomatine initiates a reactive oxygen species (ROS) burst followed by programmed cell death in *Fo* [93]. Most tomato pathogens are tolerant to alpha-tomatine, including *Fol* and *Vaa* [90]. These pathogens secrete enzymes, called tomatinases, which degrade alpha-tomatine. *Vaa* deglycosylates alpha-tomatine into the less toxic β_2_-tomatine, while *Fol* cleaves it into tomatidine and lycotetraose [90,94]. These last two compounds are not only less toxic to fungi than alpha-tomatine, but have also been implicated in the suppression of plant defense responses [95]. In total, five putative tomatinase genes have been identified in *Fol* [33]. Deletion of one of them, *TOM1*, decreased tomatinase activity by 25% and led to the formation of β_2_-tomatine instead of tomatidine. Tomato plants infected with *TOM1* deletion strains showed delayed disease symptoms, while strains overexpressing the gene showed accelerated symptom development. It will be interesting to see whether the other putative tomatinase genes of *Fol* also contribute to virulence.

To protect themselves against fungal pathogens, plants secrete chitinases that can hydrolyze chitin in fungal cell walls and can have high anti-fungal activity [96,97,98]. Fungi, on the other hand, have evolved several mechanisms to overcome this defense barrier. One of these is the secretion of the previously discussed LysM effectors, another is the secretion of proteolytic enzymes that target chitinases. Secreted protein extracts from both *Fo* and *Vd* are capable of cleaving extracellular tomato chitinases in an *in vitro* assay, showing that at least some vascular wilt fungi use this last strategy [34]. Another *in vitro* assay revealed that tomato chitinases cleaved by *Fo*-secreted proteins are reduced in their chitinase activity, showing that cleavage affects their function [34]. The observed reduction of chitinase activity was traced back to the combined activity of two secreted proteases: the metalloprotease Mep1 and the serine protease Sep1. While deletion of *MEP1* did not affect virulence of *Fol*, inoculation with a *Fol SEP1* deletion strain resulted in tomato plants that were less stunted and had a higher weight compared to control plants. Inoculation with a *mep1*/*sep1* double mutant affected not only plant weight, but also reduced other disease symptoms. Together, these data show that metallo- and serine proteases can be virulence factors. Homologs of *MEP1* and *SEP1* have been identified in the *Vd* genome and it will be interesting to see whether these are also required for full virulence. Furthermore, these experiments show that the virulence activity of a protein can be overlooked in single deletion mutants.

Plant hormones play an important role in disease resistance and components of hormonal pathways are known to be pathogen targets [99]. Pathogens can, for example, secrete effectors that target plant enzymes involved in these pathways, produce (mimics of) phytohormones or secrete proteins with enzymatic activity affecting hormone production. An example of the last category is an isochorismatase, Isc1, secreted by *Vd* to manipulate host salicylic acid (SA) biosynthesis by converting isochorismate into 2,3-dihydroxybenzoate (DDHB) [35]. Although *Vd* Isc1 lacks a canonical N-terminal signal peptide for secretion, the protein has been found in *Vd* culture supernatant by western blotting and is suggested to be non-classically secreted. *Vd* Isc1 is a virulence factor, because *Vd ISC1* deletion strains show reduced disease symptoms on cotton and *Arabidopsis* plants, compared to wild-type or complemented strains. This virulence activity is dependent on the enzymatic activity of Isc1, as proteins mutated in isochorismatase catalytic residues are unable to complement the reduced virulence phenotype observed in gene deletion strains. By converting isochorismate into DDHB, *Vd* Isc1 may reduce the conversion of isochorismate to SA to enhance susceptibility.

## 6. Hydrophobins

Filamentous fungi secrete small (~100 amino acids) proteins that contain eight cysteine residues and are capable of self-assembly into an amphiphatic membrane. Due to their water repellent properties they are called hydrophobins. They have been implicated in several processes, including surface attachment, the formation of aerial structures and the dispersal of reproductive structures [100,101].

The hydrophobin cerato-ulmin (CU), secreted by the Dutch elm disease agent *Ophiostoma*, has been well studied. More virulent *Ophiostoma* strains secrete more CU, suggesting that the protein plays a role in pathogenicity [10,102]. Furthermore, injecting purified CU into elm trees causes typical Dutch elm disease symptoms [102]. However, deleting the *CU* gene in a highly aggressive *Onu* strain did not affect virulence [36] and neither did overexpressing an *Onu CU* gene in a non-aggressive *Ou* strain [103]. Gene manipulation in these strains did result in morphological changes: the *CU* deletion strain had an “easily wettable” phenotype, while the overexpressing strain formed more aerial hyphae. Surprisingly, introduction of the *Onu CU* gene into *Ophiostoma quercus*, a related sap-staining fungus on hardwoods, enabled several independent strains to infect elm trees and cause Dutch elm disease symptoms, although to a lesser extent than the control *Onu* strain [104]. Thus, it is currently debatable whether CU contributes to virulence of the Dutch elm disease pathogen. It has been proposed that CU production enhances natural infection by promoting the binding of infectious propagules to beetles and by forming a protective layer around them during transit, thereby increasing the amount of infectious propagules that reach a new host tree [103].

Another hydrophobin, VDH1, was identified in *Vd* and has a homologue in *Vaa* [37]. Deletion of *VDH1* did not reduce *Vd* symptom development on tomato plants, but did severely reduce microsclerotia formation and desiccation tolerance of conidia [37]. These data suggest that VDH1 does not play a direct role in virulence, but is a possible *Vd* fitness factor that enables pathogen persistence in the soil and spread of the disease.

## 7. Secreted Proteins, Giveaways to the Plant Immune System

Besides passive barriers, plants have developed an active immune system to protect themselves against pathogens. The active immune system of plants is an innate, receptor-based recognition system and has traditionally been divided into two layers. In the first layer, membrane-associated Pattern Recognition Receptors (PRRs) trigger plant defense upon recognition of Pathogen-Associated Molecular Patterns (PAMPs, sometimes more accurately called MAMPs for Microbe-Associated Molecular Patterns), which are defined as highly conserved molecules that are essential for microbial fitness and common to entire classes of microbes [105,106]. This first layer is known as PAMP-triggered immunity (PTI) and triggers ROS bursts, the activation of protein kinases and massive transcriptional reprogramming. It is considered a broad-defense response, effective against a wide range of invading microbes. However, pathogens (and endophytes) secrete proteins known as effectors that are capable of manipulating plant processes to promote colonization, for example by suppressing PTI [107,108]. These effectors can be, directly or indirectly, recognized by R proteins resulting in Effector-Triggered Immunity (ETI). ETI often leads to a Hypersensitive Response (HR), a form of localized cell death [109]. Most R proteins are intracellular receptors that contain a NB-LRR domain with either an N-terminal CC or a Toll and Interleuking-1 Receptor (TIR) region [107,108]. In comparison to PTI, ETI is generally a stronger defense response. The defense response triggered by extracellular effectors secreted by foliar fungal pathogens, such as *Cladosporium fulvum* and *Leptosphaeria maculans*, does not completely fit the criteria of PTI or ETI. Hence, the term effector-triggered defense (ETD) was recently introduced to describe this resistance response [110]. ETD is initiated by membrane-localized RLPs and requires the RLK Suppressor Of BIR1-1 (SOBIR1) for downstream signaling. Compared to ETI, the resistance response is much slower and the pathogen is not eliminated, but only halted. As it is not always straightforward to distinguish between PTI and ETI it has been suggested to see them as a continuum, as reviewed in [111]. Plant immune responses against vascular wilt fungi do not usually include a HR, but instead involve callose deposition, the production of secondary metabolites and the formation of tyloses, gels and gums in the xylem vessels to prevent spreading of the pathogen [112,113]. Below, some cloned and characterized receptor proteins involved in resistance against wilt fungi are described (Table 2). As for other types of resistance based on effector recognition, the secreted proteins that these receptor proteins recognize are known as Avr proteins.

**Table 2:** Receptor proteins involved in resistance against vascular wilt fungi.

Locus	Type	Source	Avr	Reference
*Fom-1*	TIR-NB-LRR	melon cultivar Doublon		[114]
*Fom-2*	NB-LRR	melon cultivar CM17187	AvrFom2	[115]
*Fom-4* *		melon cultivar Tortuga		[116]
*I*		wild tomato *S. pimpinellifolium*	Avr1/Six4	[117]
*I-1*		wild tomato *S. pennellii*	Avr1/Six4	[118]
*I-2*	CC-NB-LRR	wild tomato *S. pimpinellifolium*	Avr2/Six3	[57]
*I-3*	SRLK	wild tomato *S. pennellii*	Avr3/Six1	[48]
*I-4*		*S. lycopersicum*		[119]
*I-5*		wild tomato *S. pennellii*		[119]
*I-6*		wild tomato *S. pennellii*		[119]
*I-7*	LRR-RLP	wild tomato *S. pennellii*		[56]
*RFO1*	WAKL-RLK	*Arabidopsis thaliana* ecotype Col-0		[120]
*RFO2*		*Arabidopsis thaliana* ecotype Col-0		[121]
*RFO3*	SRLK	*Arabidopsis thaliana* ecotype Col-0		[122]
*RFO4*		*Arabidopsis thaliana* ecotype Col-0		[120]
*RFO5*		*Arabidopsis thaliana* ecotype Col-0		[120]
*RFO6*		*Arabidopsis thaliana* ecotype Col-0		[120]
*RFO7*		*Arabidopsis thaliana* ecotype Col-0		[123]
*Ve1*	LRR-RLP	tomato cultivar Craigella	Ave1	[70,124]
*GbVe*	LRR-RLP	cotton cultivar Pima90-53		[125]

* = recessive resistance gene; I = immunity; RFO = resistance to *Fusarium oxysporum*; *Gb* = *Gossypium barbadense*; TIR = toll and interleukin-1 receptor; NB = nucleotide-binding; LRR = leucine-rich repeat; CC = coiled-coil; SRLK = S-receptor-like kinase; RLP = receptor-like protein; WAKL = wall-associated kinase-like; S. = solanum; Avr = avirulence; Six = secreted in xylem.

Intracellular R proteins that confer resistance to *Fo* have been found in melon (Fom-1 and Fom-2) and tomato (I-2). Fom-1 confers resistance to *Fo* f. sp. *melonis* (*Fom*) races 0 and 2. A map-based cloning strategy identified the gene and sequence analysis showed it encodes a TIR-NB-LRR [114]. Future studies should provide functional validation of the gene and identify the corresponding *AVR* gene in *Fom*. *Fom-2* provides resistance against *Fom* races 0 and 1. It was also identified by map-based cloning and encodes a NB-LRR protein that does not contain an N-terminal TIR or CC domain [115]. Recently, *Fom AVRFOM2* was identified by comparative genomics between *Fom* strains of different races [126]. The gene is highly induced upon melon infection and encodes a small, secreted protein of 167 amino acids that contains two cysteine residues and no recognizable motifs. Tomato *I-2* encodes a classical CC-NB-LRR protein that is mainly expressed in the vascular tissue surrounding xylem vessels [57,113,127]. I-2 recognizes the small, cysteine-rich protein Avr2 (six cysteines in a 22 kDa mature protein), also known as Six3 [49]. However, the presence of both *Fol* Avr2 and *Fol* Six5 is required to trigger I-2 mediated immune responses in tomato plants during infection [51]. Because Six3 alone is sufficient to induce an I-2-dependent HR in a heterologous system and single amino acid changes in Six3 suffice to prevent recognition by I-2, this protein is called Avr2 and Six5 is not [49,51].

Recently, two other tomato genes that confer resistance to *Fol* were identified. The first one, *I-3*, is already employed as a resistance gene in cultivated tomato and recognizes *Fol* Avr3, also known as Six1 [44]. Map based-cloning experiments, followed by transgenic complementation assays, have shown that I-3 is an SRLK [48]. Because I-3 is localized in the plasma membrane, it is assumed that the I-3 ectodomain recognizes Avr3. This suggests that Avr3 is an apoplastic effector, but does not exclude uptake of Avr3 into plant cells. Future research should indicate whether I-3 recognizes Avr3 directly, although no interaction was found in a yeast-two-hybrid assay [48], or indirectly by monitoring perturbation of plant processes. A cell death response has never been observed upon co-expression of Avr3 and I-3 [48] or expression of *AVR3* in I-3 plants, either stably or transiently [47,48], in contrast to Avr2 and I-2 [49]. The other recently identified tomato resistance gene is *I-7*. RNA-sequencing and single nucleotide polymorphism analysis were used to identify *I-7* as a LRR-RLP [56]. Like I-3, I-7 also confers resistance to *Fol* race 3 strains (as well as to race 1 and 2). However, it does not seem to recognize Avr3 and it is currently unknown which effector protein it does recognize.

Two homologous LRR-RLPs in tomato and cotton, Ve1 and GbVe1, have been identified that confer resistance to Verticillium wilt [70,124,125,128]. Tomato Ve1 confers resistance against race 1 isolates by directly or indirectly recognizing Ave1, a secreted protein with homology to plant PNPs [26]. Homologs of *AVE1* have been identified in the bacterial plant-pathogen *Xanthomonas axonopodis* (*Xac*) and in several fungal species, including *Fo*. Hence, it was hypothesized that *Ve1* might also confer resistance to *Fo*. *Ve1* expressing tomato plants were indeed reported to confer resistance to *Fo* [26]. However, this observation could not be confirmed in another lab [129], possibly because *FoAVE1* was not expressed *in planta* [6]. Interestingly, tomato *Ve1* can be transferred to *Arabidopsis thaliana* (Arabidopsis) and retain its ability to confer resistance against race 1 isolates of *Vd* and *Vaa* [130].

Some pathogenic strains of *Fo* isolated from related crucifer hosts can produce disease symptoms on Arabidopsis [120]. Differential susceptibility between two Arabidopsis ecotypes to *Fo* f. sp. *matthioli* was used to identify six dominant, quantitative resistance loci by map-based cloning [120]. These loci were named Resistance to Fusarium Oxysporum 1-6 (*RFO1-6*). So far, three *RFO* genes have been identified. *RFO1* encodes a RLK that contains an extracellular wall-associated kinase-like (WAKL) domain [120]. Resistance conferred by *RFO2*, *RFO4* and *RFO6* is dependent on the presence of *RFO1*. Interestingly, a *rfo1* mutant is more susceptible to several crucifer-specific *formae speciales*, suggesting it might play a role in basal defense [120]. *RFO2* is an LRR-RLP [121]. The extracellular LRRs in RFO2 are very similar to the LRRs in the RLK PSY1R. PSY1R perceives the tyrosine-sulfated peptide PSY1 that is secreted by plant cells and is involved in plant growth, development and defense [131]. Hence, a decoy model has been proposed in which *Fo* secretes an effector that targets PSY1R to enhance plant susceptibility, but in the presence of RFO2 the effector is recognized and resistance responses are induced instead [121]. *RFO3* is a SRLK, like the tomato *I-3* gene, and confers quantitative resistance to *Fo* f. sp. *matthioli*, but not against two other crucifer-specific *formae speciales* [122]. *RFO3* expression is highest in the vasculature and its expression in the root is required for enhanced disease resistance and reduced colonization. The *Fo* derived signal recognized by RFO3 has not yet been identified, but is expected to be extracellular since RFO3 is an SRLK.

The above examples show that plants deploy at least three different types of receptor proteins (RLPs, RLKs and NB-LRRs) to trigger defense responses against *Fo*. It will be interesting to see whether plant defenses against other vascular wilt fungi are as varied. For none of the receptors described in this review it is currently known whether they directly recognize a secreted protein or compound or whether they monitor pathogen-induced changes in plant processes. It has been shown that defense responses triggered by I-7 and Ve1, both LRR-RLPs, are dependent on EDS1 [56,70], a positive regulator of basal defense responses that is also required for TIR-NB-LRR mediated resistance [132]. Otherwise, little is known about the downstream processes that take place after these receptors are activated. Although Ve1-resistance is not mediated against a foliar pathogen, it does fit the criteria for ETD [110]. One of these criteria is that the pathogen is not eliminated from the plant, but that spreading is prevented. It is seen more often that vascular wilt fungi are able to colonize a resistant plant, although markedly reduced compared to a susceptible plant [133,134].

## 8. Concluding Remarks

This review shows that vascular wilt fungi secrete many different types of proteins to manipulate their hosts and enhance disease susceptibility. Although some gene families are expanded in *Vd*, *Vaa* and *Fo*, it is clear that the ability to colonize xylem is not due to shared (*i.e.*, homologous) virulence factors and has therefore likely arisen independently several times in fungal evolution. Discovery of a virulence function for a secreted protein can be hampered by functional redundancy, in which case testing of a multi-gene deletion strain is paramount, but not always feasible. Furthermore, the effect of a pathogen-secreted protein can depend on its host. To better understand pathogen–host interactions it is key to not only identify virulence factors, but also functionally characterize them. Conserved domains and homology to proteins with a known function are absent for many small, cysteine-rich proteins. The most promising avenue, then, to find a starting point to unravel the function of a pathogen-secreted protein is the identification of plant targets. This could lead to the discovery of new susceptibility (*S*) genes, recessive genes required for pathogen infection, which can offer an alternative to *R* genes in resistance breeding [135].
